# PCOSKB_R2_: a database of genes, diseases, pathways, and networks associated with polycystic ovary syndrome

**DOI:** 10.1038/s41598-020-71418-8

**Published:** 2020-09-07

**Authors:** Mridula Sharma, Ram Shankar Barai, Indra Kundu, Sameeksha Bhaye, Khushal Pokar, Susan Idicula-Thomas

**Affiliations:** Biomedical Informatics Center, Indian Council of Medical Research-National Institute for Research in Reproductive Health, Mumbai, 400012 India

**Keywords:** Databases, Reproductive disorders, Gene regulatory networks

## Abstract

PolyCystic Ovary Syndrome KnowledgeBase (PCOSKB_R2_) is a manually curated database with information on 533 genes, 145 SNPs, 29 miRNAs, 1,150 pathways, and 1,237 diseases associated with PCOS. This data has been retrieved based on evidence gleaned by critically reviewing literature and related records available for PCOS in databases such as KEGG, DisGeNET, OMIM, GO, Reactome, STRING, and dbSNP. Since PCOS is associated with multiple genes and comorbidities, data mining algorithms for comorbidity prediction and identification of enriched pathways and hub genes are integrated in PCOSKB_R2_, making it an ideal research platform for PCOS. PCOSKB_R2_ is freely accessible at http://www.pcoskb.bicnirrh.res.in/.

## Introduction

Polycystic ovary syndrome (PCOS) is the most common endocrine disorder in women of reproductive age^1^. The syndrome encompasses a broad spectrum of signs and symptoms, making the diagnosis of PCOS challenging. There exist many society-based guidelines for PCOS diagnosis such as the (i) Rotterdam criteria accepted by European Society for Human Reproduction and Embryology(ESHRE) and American Society for Reproductive Medicine (ASRM)^2^; (ii) National Institutes of Health or National Institute of Child Health and Human Disease (NIH/NICHD) criteria^3^ and (iii) Androgen Excess and PCOS Society (AE-PCOS/AES) criteria^4^. These guidelines rely on the presence of oligo-anovulation and hyperandrogenism, after excluding other androgen excess or related disorders, for diagnosis of PCOS. The prevalence of PCOS globally ranges from 2.2 to 26% contingent upon the population assessed and the criteria used for evaluation^5^. Many of the women with PCOS suffer from various comorbid conditions such as glucose intolerance^6^, type-II diabetes^7^, cardiovascular ailments^8^, anxiety disorders^9^, bipolar disorders^10^ and sleep-related disorders^11^.

The increasing prevalence of PCOS and its profound impact on the physical and mental health of women has catapulted research efforts to elucidate the genetic etiology and pathophysiology of PCOS^12^. This, in turn, has led to a surge in PCOS-related data available in the public domain; calling for an urgent need to manually curate and collate this information as online databases for researchers and clinicians.

The databases dedicated to PCOS, currently available online are PCOSKB^13^ and PCOSBase^14^. As on date, PCOSDB^15^ is not accessible. PCOSBase, categorized as a manually curated database, lists 8,185 proteins as associated with PCOS. This data is a compilation from 9 databases and 30 published expression studies, without having stringent criteria for cataloguing a protein as “PCOS-related”. PCOSKB, developed by our group in 2015, was created by critically reviewing the scientific literature available for PCOS. The manual curation exercise resulted in a list of 241 genes, which was further linked with relevant molecular, biochemical, and clinical data along with supporting reference literature.

Over the past 5 years, there has been a significant increase in the data available on PCOS. Here, we present an update to the content and functionality of the PCOSKB database. PCOSKB_R2_ holds information of 533 genes and 29 miRNAs (manually curated) identified from peer-reviewed literature, based on experiments such as RT-PCR, western blotting, immunochemistry, and cell-based assays. Additionally, information on 4,023 genes identified from microarray expression studies on PCOS is also included in PCOSKB_R2_. The PCOS genes are further linked with gene ontology terms, pathways, diseases, and SNPs.

Besides retrieving data, researchers can analyse the data in PCOSKB_R2,_ using various tools embedded in the database such as *Comorbidity analysis* for estimating the risk of diseases to co-occur with PCOS; *Network analysis* for identifying enriched pathways and hub genes and *Venn analysis*^16^ for finding common and unique genes, pathways and ontologies. PCOSKB_R2_ will enable researchers and clinicians to efficiently interrogate the published data on PCOS and identify gaps in our current understanding of PCOS and its comorbidities.

## Results and discussion

PCOSKB_R2_ was developed using PHP 7.2.24, MariaDB Server 10.1.44, JavaScript, AnyChart 8.7.1, vis.js 4.21, R version 3.6.3 and XHTML 1.0. It has client server-based architecture and is hosted on Apache webserver 2.4.29 with a Linux environment.

**Figure 1:**
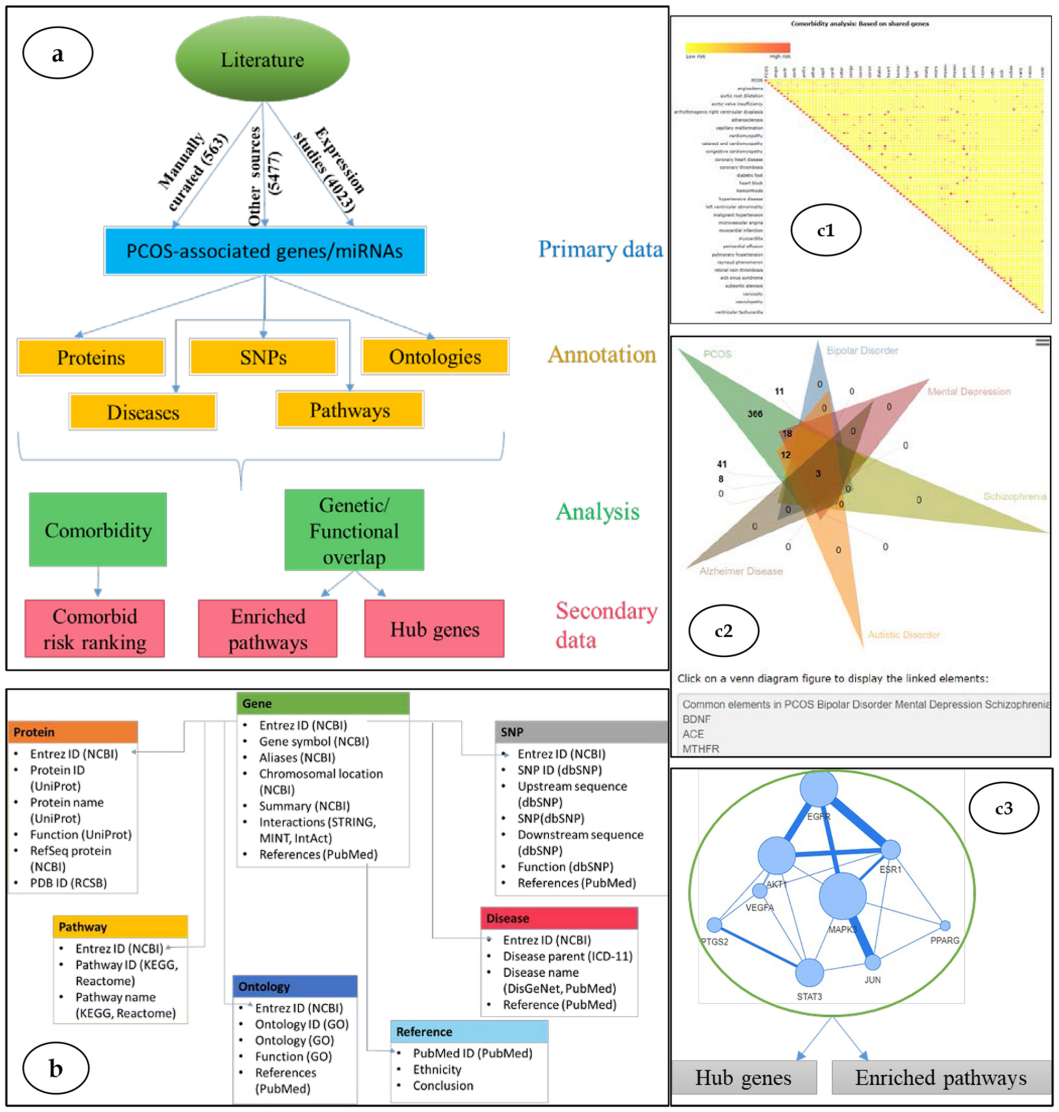
Conceptual and relational view of data and tools in PCOSKB_R2_.

PCOSKB_R2_ has an interactive and user-friendly interface. The homepage provides a short description of the database and its functionalities. The data is organized into datasets dedicated to (a) genes, (b) miRNAs, (c) SNPs, (d) diseases, (e) pathways, and (f) gene ontology terms associated with PCOS (Fig. 1a,b). These datasets can be easily accessed using the navigation tabs located on the top panel of the webpage. A brief description of these tabs is given below:

- *Search*
  - *Quick search* enables users to retrieve information based on keywords; all the information available in PCOSKB related to the keyword is displayed.
  - *Advanced search* enables users to build specific queries for a gene, protein, SNP, miRNA, diseases, or pathways associated with PCOS.
- *Browse* This tab enables users to surf the datasets for genes, miRNAs, SNPs, diseases, pathways, and gene ontology terms associated with PCOS.
- *Tools* Algorithms for comorbidity, network, and Venn analysis can be accessed here.
  - *Comorbidity Analysis* This tool can be used to predict comorbidity for selected diseases based on (i) shared genes, (ii) uniqueness of shared genes, (iii) shared ontologies, and (iv) network-based separation of shared genes (Fig. 1c1). The results for each of these modules can be downloaded as heatmap images (colored based on comorbidity scores) and spreadsheets with detailed information on shared genes and pairwise comorbidity scores for the selected diseases.
  - *Network analysis* The tool provides a disease-disease network for selected diseases, the enriched pathways in these diseases, and the hub and bottleneck genes that are critical for these diseases (Fig. 1c3). The results can be downloaded as spreadsheets or images.
  - *Venn analysis* This tool can be used to illustrate the unique and/or common genes, pathways, and ontologies for 2 or more (up to 6) diseases (Fig. 1c2). The analysis can be downloaded as Venn images or spreadsheets.
- *Help*: This page provides detailed information, with examples, for efficiently navigating the PCOSKB interface and using the data-mining algorithms.

The applications of these datasets and algorithms for estimating the comorbidity risk and understanding the genetic and functional overlap in comorbid conditions of PCOS are demonstrated by case studies.

A. Estimation of comorbidity risk:

**Case 1:** *PCOS, Diabetes, and Hypertension.*

There is ample clinical evidence that women with PCOS are more likely to suffer from diabetes and hypertension as compared to other cardiac ailments^17–20^.

**Figure 2:**
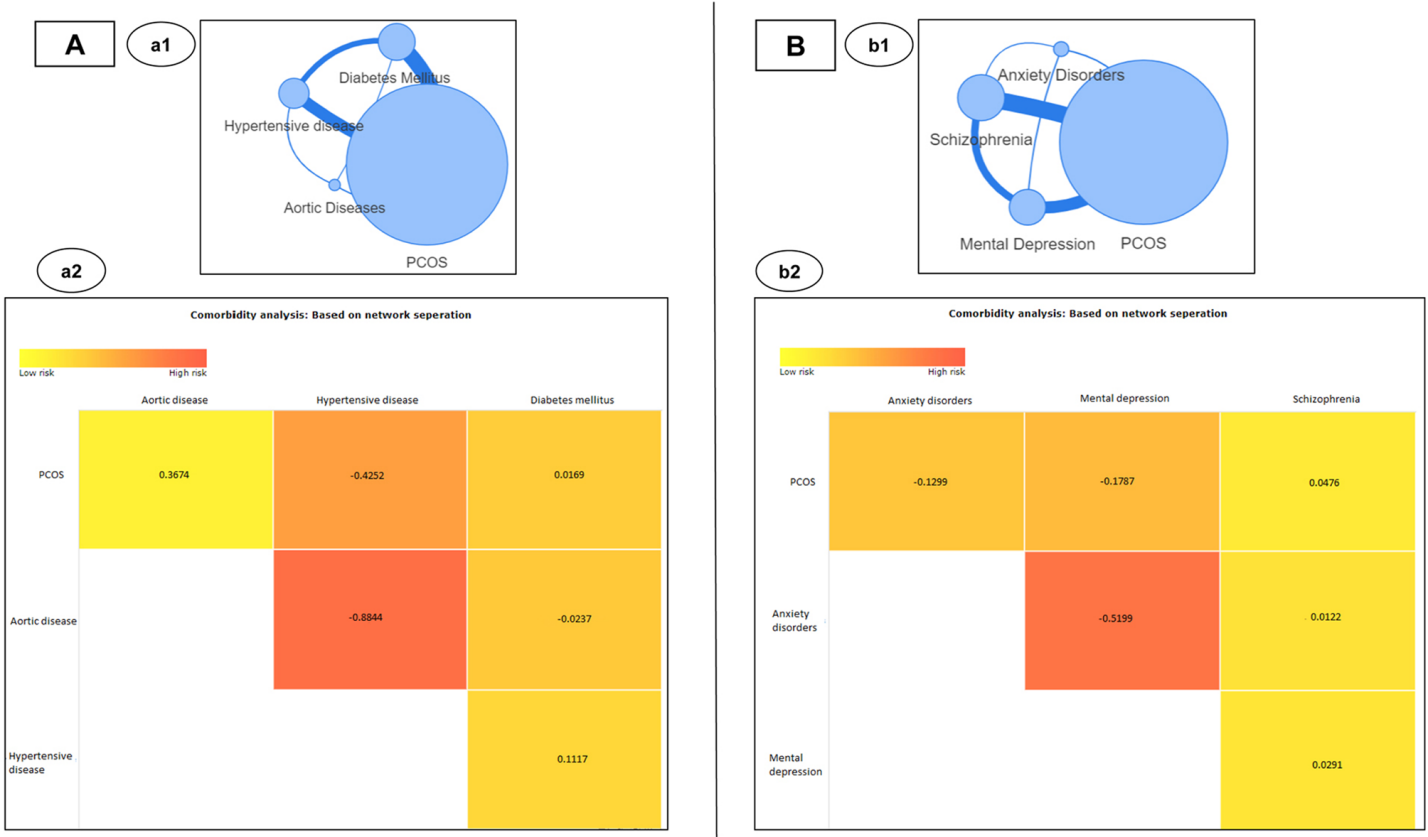
Network-based comorbidity analysis for PCOS and (**A**) diabetes and hypertension; (**B**) psychological disorders.

The comorbidity risk can be estimated using the ‘*Comorbidity analysis*’ algorithm in PCOSKB_R2_. In accordance with the clinical reports, when disease terms such as diabetes mellitus, hypertensive diseases along with a less frequently observed comorbidity such as aortic diseases were analyzed for comorbidity scores; it was found that the risk for diabetes and hypertensive diseases to co-occur with PCOS was much higher as compared to aortic diseases. Expectedly, the maximum comorbidity score amongst the selected diseases was found to be between aortic diseases and hypertension (Fig. 2A). The above example illustrates the utility of the comorbidity analysis algorithm for estimating the risk of diseases to co-occur in PCOS.

**Case 2:** *PCOS and Psychological disorders*.

Women with PCOS are known to have an increased risk (albeit at varying levels) of suffering from mental health conditions such as anxiety, depression, and schizophrenia^21,22^. A study by Rassi et al., concluded that 57% of women with PCOS are diagnosed with at least one of the psychiatric disorders^23^. In an ambulatory population of 72 women with PCOS, it was observed that mental depression and schizophrenia were the most and least prevalent respectively among the psychiatric disorders^23^. Through a population-based retrospective study in a cohort of 5,431 women with PCOS and 21,724 controls, a significantly higher incidence of depressive and anxiety disorders were reported in women with PCOS^24^. In another study, the prevalence of psychiatric comorbidity and depression was reported as the most common disorder in women with PCOS followed by anxiety^25^. Meta-analysis of 57 studies (172,040 patients) summarised that women with PCOS were most likely to get diagnosed with depression followed by anxiety^26^.

These clinical observations were accurately captured through the comorbidity scores generated using the network-based separation method. Mental depression had the highest comorbidity risk followed by anxiety disorders and schizophrenia (Fig. 2B). It is noteworthy that although maximum number of genes (124) overlapped between PCOS and schizophrenia, as reflected in the edge thickness between these 2 disease nodes; comorbidity analysis correctly estimated the least risk for comorbidity with schizophrenia amongst the three mental diseases, in accordance with literature reports; highlighting the predictive power of network-based separation method for comorbidity analysis.

B. Identification of the genetic and functional overlap in comorbid conditions.

**Case 1:** *PCOS, Diabetes, and Hypertension.*

Although, diabetes and hypertension are commonly observed comorbid conditions in women with PCOS; not much is known about the genetic overlap of these disorders^27^.

Venn analysis revealed that 32 genes and 364 pathways are commonly associated with PCOS, diabetes, and hypertension (Supplementary Table S1). Network analysis identified 104 enriched pathways, 21 hub genes, and 10 bottleneck genes for these diseases (Supplementary Figs. S1a1 and S1a2, Supplementary Table S1). Hub genes, due to their high degree of inter-cluster connectivity, play an important role in the crosstalk of enriched pathways. We mined literature for ascertaining the association of these 21 genes with the comorbid conditions of diabetes, hypertension, and PCOS. Of the 21 genes, we found literature evidence for association of four genes (*ESR1*, *PTGS2, LEP, PPARG)* with these comorbidities, as detailed below.

(i) *ESR1* codes for estrogen receptor alpha and hence *ESR1* mutations can increase the risk of estrogen-dependent pathophysiologies. In a study by Zhao L et al., *ESR1* polymorphisms were reported to be associated with hypertension and diabetes^28^. A case–control study by Jiao X et al., documented that altered expression of *ESR1* can influence the risk of PCOS and its upregulation may contribute to abnormal follicular development^29,30^.
(ii) Prostaglandin-endoperoxide synthase (*PTGS2)* is a key enzyme for biosynthesis of the inflammatory hormone prostaglandin. It is known to be upregulated in granulosa cells of women with PCOS and arteries of patients with hypertension and diabetes^31,32^.
(iii) Leptin hormone encoded by the leptin gene (*LEP)* plays an important role in the regulation of energy homeostasis and body weight management. Several independent studies have reported the association of leptin receptor deficiency in diabetes, hypertension, and PCOS. High circulatory leptin has been observed in patients with a cluster of metabolic syndrome including hypertension, diabetes^33^, and PCOS^69^.
(iv) Peroxisome proliferator-activated receptor gamma (*PPARG)* regulates adipocyte differentiation and thereby controls beta-oxidation of fatty acids. Mutations in *PPARG* are known to increase the risk for development of hypertension and diabetes^34^.

In addition to the identification of hub and bottleneck genes, the *View interaction* option in the *Gene network analysis* tool can be used to display the tissue-specific interacting partners of each gene in the network (Supplementary Fig. S1). Using this feature, we identified two genes (*PON1, ADIPOQ)* that interact with multiple hub genes (Supplementary Figs. S1a3 and S1a4). *PON1* interacts with six hub genes (*TNF*, *IL6*, *INS*, *CCL2*, *LEP*, *PPARG*) and one bottleneck gene (*LIPC*) (Supplementary Fig. S1a4). Adiponectin *(ADIPOQ*) interacts with 19 hub genes that are expressed in adipose tissue (Supplementary Fig. S1a3). The association of both these genes in the comorbid conditions of type 2 diabetes, hypertension, and PCOS is documented in the literature. Paraoxonase-1 (*PON1*) mediates enzymatic protection of low-density lipoprotein (LDL) against oxidative modifications and is known to be associated with diabetes, hypertension, and PCOS^35,36^. Low levels of adiponectin are associated with several obesity-related disorders^37^ and *ADIPOQ* is a biomarker for type-2 diabetes, hypertension^38^, and PCOS^39^.

This case study illustrates the utility of the *Gene network analysis* tool in deciphering the genetic and functional overlap of comorbid conditions. While the role of all the identified hub genes in PCOS, diabetes, and hypertension individually has been well established, it would be worthwhile to establish the role of these hub genes in the pathophysiology of PCOS, diabetes, and hypertension, as a combined disease state, and explore them as polypharmacological drug targets.

**Case 2:** *PCOS and Psychological disorders—anxiety and mental depression.*

Insulin resistance, obesity, and altered levels of androgens (Supplementary Table S2) have been reported as the common pathophysiological link between PCOS and psychiatric disorders^24,40^. Interestingly, evaluation of enriched pathways for the top two psychological disorders (mental depression and anxiety) that are comorbid with PCOS revealed pathways that represent these cellular mechanisms (Supplementary Table S2, Supplementary Figs. S1b1 and S1b2, Supplementary Table S1).

Network analysis of the enriched pathways revealed 21 hub genes and 10 bottleneck genes. Of these, the role of two hub genes (*IL6*, *STAT3*) in the comorbidity of PCOS and selected psychiatric disorders has been reported in literature. Kawamura S et al., reported elevated levels of inflammatory cytokine *IL6* in women suffering from PCOS and depression^41^. The negative association of *STAT3* with anxiety and depression have been reported by Feng and Shao in PCOS induced rat models^42^. Anxiety and depression in rats were analysed based on their decreased locomotor activity in behavioural tests such as open-field tests, object recognition tests, and elevated plus maze tests.

**Case 3:** *PCOS and Pregnancy-related disorders—preeclampsia.*

Women with PCOS are known to be at higher risk of pregnancy-related disorders as compared to women without PCOS^43,44^. In PCOSKB, genes, and miRNAs associated with pregnancy-related disease terms like “Pregnancy complications, Cardiovascular”, “Pregnancy associated hypertension”, “Ectopic pregnancy”, “Gestational diabetes”, and “Preeclampsia” can be accessed under the disease category of reproductive disorders.

miRNAs are known to play a critical role in the pathogenesis of PCOS and pregnancy-related disorders^45–47^. Pathways such as adipocytokine signaling, oxytocin signaling, TNF signaling, progesterone-mediated oocyte maturation, estrogen signaling, MAPK, and FoxO signaling are known to be regulated by miRNAs and associated with pregnancy outcome^48,49^.

miRNA-based pathway enrichment analysis of preeclampsia revealed 88 enriched pathways that included progesterone-mediated oocyte maturation, estrogen signaling, MAPK signaling, and FoxO signaling pathways (Supplementary Table S1); these pathways are known to be associated with PCOS and preeclampsia in literature^49–51^.

## Conclusion and future directions

The aim of developing PCOSKB_R2_ was to provide a one-stop online portal for accessing manually curated information on PCOS to the community of clinicians and researchers. The genes, listed in the manually curated dataset of PCOSKB_R2_ were identified based on the inference and data mined from publications. Relevant annotations of these genes such as gene interactions, pathway associations, and SNPs have been provided along with links to the reference literature.

This second release of PCOSKB has substantial advancement both in terms of data and analysis tools^13^. In addition to the advanced search and browser features for efficiently interrogating the database, users can avail of the tools to predict comorbidity risks, enriched pathways, and hub genes for selected diseases. These tools are powerful for gaining insights on the comorbidities of PCOS and the underlying gene-pathway associations, as can be seen by the aforementioned case studies. However, users need to be aware and cautious of the publishing or literature bias that can lead to erroneous inferences.

**Figure 3:**
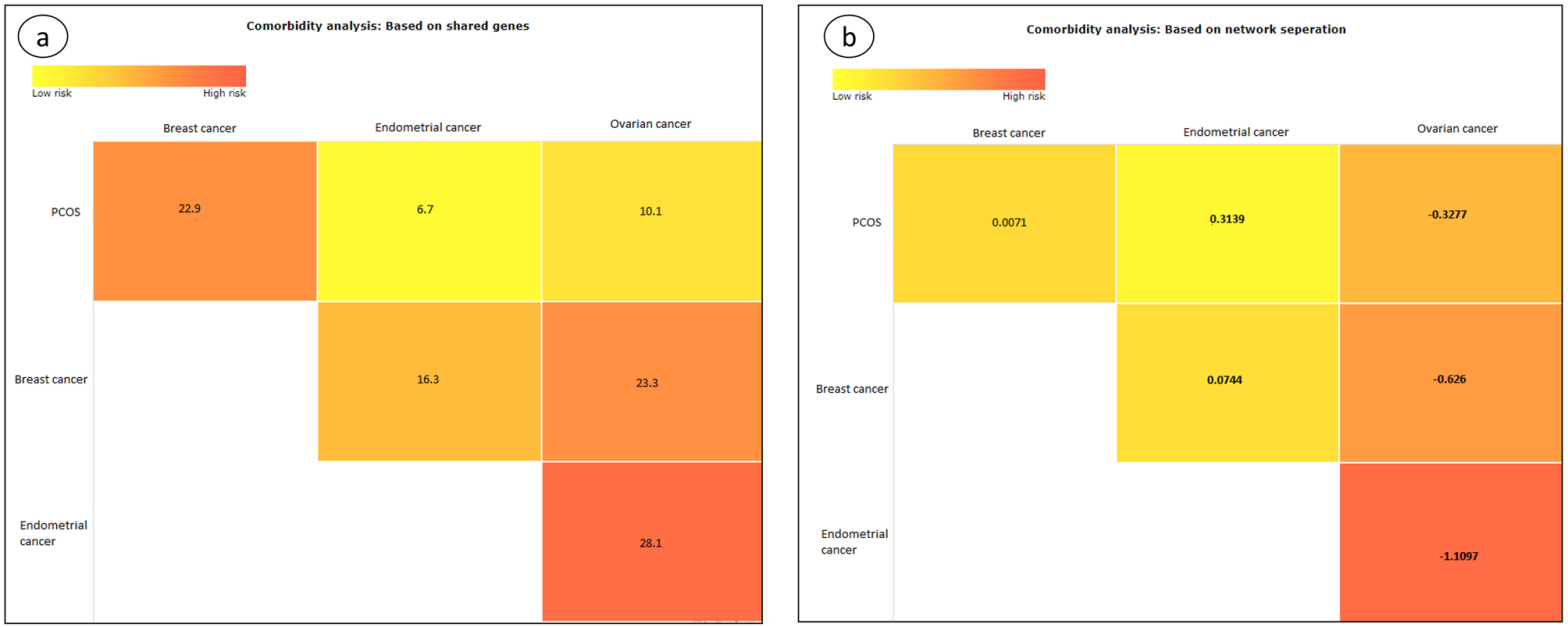
Comorbidity analysis for PCOS and cancers using (**a**) shared genes and (**b**) network-separation methods.

The impact of publication bias on the results of the comorbidity analysis tool can be assessed by the following example. Women with PCOS are known to suffer from an increased risk of endometrial cancer followed by ovarian cancer as compared to women without PCOS^50^. The incidence of breast cancer is similar in women with and without PCOS^41,50,51^. The comorbidity analysis tool, using the method of shared genes, incorrectly predicted the highest risk of comorbidity for breast, followed by ovarian and least for endometrial cancer (Fig. 3). This error is inadvertently caused due to the positive publication bias for breast cancer (407,285 PubMed records) as compared to ovarian (116,514 PubMed records) and endometrial cancers (37,950 PubMed records). Hence, the genes that are known to be associated with endometrial cancer are far lesser (38 genes) than ovarian (57 genes) and breast cancers (129 genes).

The network separation based algorithm identified the highest comorbidity risk for ovarian, followed by breast and endometrial cancers (Fig. 3). The network separation method is based on the distance/separation of the disease-causing genes in pathway networks and therefore is more robust and less dependent (not independent) on the number of disease-causing genes as compared to the algorithm of shared genes. This algorithm should, therefore, be the choice for comorbidity prediction when a fewer number of diseases; with possibility for publication bias is analysed.

The incidence of PCOS is rising globally^52–56^ and we expect the data, generated on PCOS, to increase exponentially in the years to come. Depending on the availability and nature of data generated from these research efforts, PCOSKB_R2_ will be updated with new information and analysis tools. Hopefully, with more data, the negative impact of publication bias will be reduced. PCOSKB_R2_ will be a comprehensive source of updated and curated information on gene-disease-pathway associations in PCOS and its comorbidities.

## Methods

### Dataset curation

#### Curation of the gene dataset

The genes associated with PCOS were identified by querying PubMed^57^ with MeSH(Medical Subject Headings)^58^ terms such as, “Ovary Syndrome, Polycystic”, “Syndrome, Polycystic Ovary”, “Stein-Leventhal Syndrome”, “Stein Leventhal Syndrome”, “Syndrome, Stein-Leventhal”, “Sclerocystic Ovarian Degeneration”, “Ovarian Degeneration, Sclerocystic”, “Sclerocystic Ovary Syndrome”, “Polycystic Ovarian Syndrome”, “Ovarian Syndrome, Polycystic”, “Polycystic Ovary Syndrome 1”, “Sclerocystic Ovaries”, “Ovary, Sclerocystic”, “Sclerocystic Ovary”, “PCOS” and “Gene”. Using this query, 1561 literature records were retrieved from PubMed.

The association of 533 genes with PCOS was manually confirmed by critically reviewing the 1561 publications. A gene was verified to be PCOS-associated if the literature mentions experimental evidence based on RT-PCR, western blotting, immunochemistry, and cell-based assays. Additional annotations such as nature of the study population, ethnicity, mutations/SNPs, unique identifiers for gene and protein records, protein structures, family and ontology details, metabolic pathway information were obtained from literature and mapping the gene records to databases such as Gene^59^, dbSNP^60^, Ensembl^61^, UniProt^62^, PDB^63^, GO^64^, KEGG^65^, OMIM^66^, Reactome^67^ and STRING^68^ (Supplementary Table S3).

#### Curation of the gene-disease association dataset

Disease associations of the PCOS genes were retrieved from DisGeNET^69^ and PubMed^57^ databases. The disease terms in DisGeNET that are linked to PubMed literature and have an active MedGen^70^ *ConceptID (CUI)* were retained for further curation. The terms with disease type as *“phenotype”* and disease semantic type as “finding”, “pathologic function”, “sign or symptom”, “injury or poisoning”, “experimental model of disease”, “experimental model of disease; Neoplastic process”, “anatomical abnormality”, “organism attribute” were discarded from the list as the terms under these headers did not refer to diseases.

This list was further subdivided into two sets based on the source of information in DisGeNET^69^. Dataset ‘*A*’ comprised of gene-disease associations collated in DisGeNET from manually curated databases such as ClinVar^71^, CTD^72^, Genomics England^73^, GWAS Catalog^74^ and GWAS^75^ and Dataset ‘*B*’ had information collated from text mining datasets such as BEFREE^76^ and LHGDN^77^. Since dataset ‘*A*’ records were from curated sources, these were included in PCOSKB_R2_ without further verification. For dataset ‘*B*’, gene-disease associations were validated based on rigorous manual curation. The associated literature was reviewed carefully and evidence for gene-disease association was sourced from experimental techniques involving human samples, such as RT-PCR, western blotting, immunochemistry, and cell-based assays. Genes that did not have any disease information in DisGeNET were queried in PubMed and publication records were mined using pubmed.mineR package^78^.

In cases, wherein multiple disease terms referred to the same disease, the terms were retitled as explained in Table 1.

**Table 1:** Rules for redundancy elimination in gene-disease association dataset.

S. No	Types of redundancy	Examples	
		Disease terms	Modified term
1	Target organ of disease	‘Malignant neoplasm of ovary’, ‘ovarian neoplasm’, ‘Epithelial ovarian cancer’	Ovarian cancer
2	Age of onset of disease	‘Adult type dermatomyositis’, ‘Dermatomyositis, Childhood Type’,‘Dermatomyositis’	Dermatomyositis
3	Synonyms of disease	‘Mental Depression’, ‘Major Depressive Disorder’, ‘Depressive disorder’	Mental Depression
4	Severity of disease	‘Mental disorder’, ‘Mental disorder, severe’, ‘Mental disorder, acute’, ‘mental disorder, chronic’	Mental disorder

#### Unique categorization of disease groups

**Figure 4:**
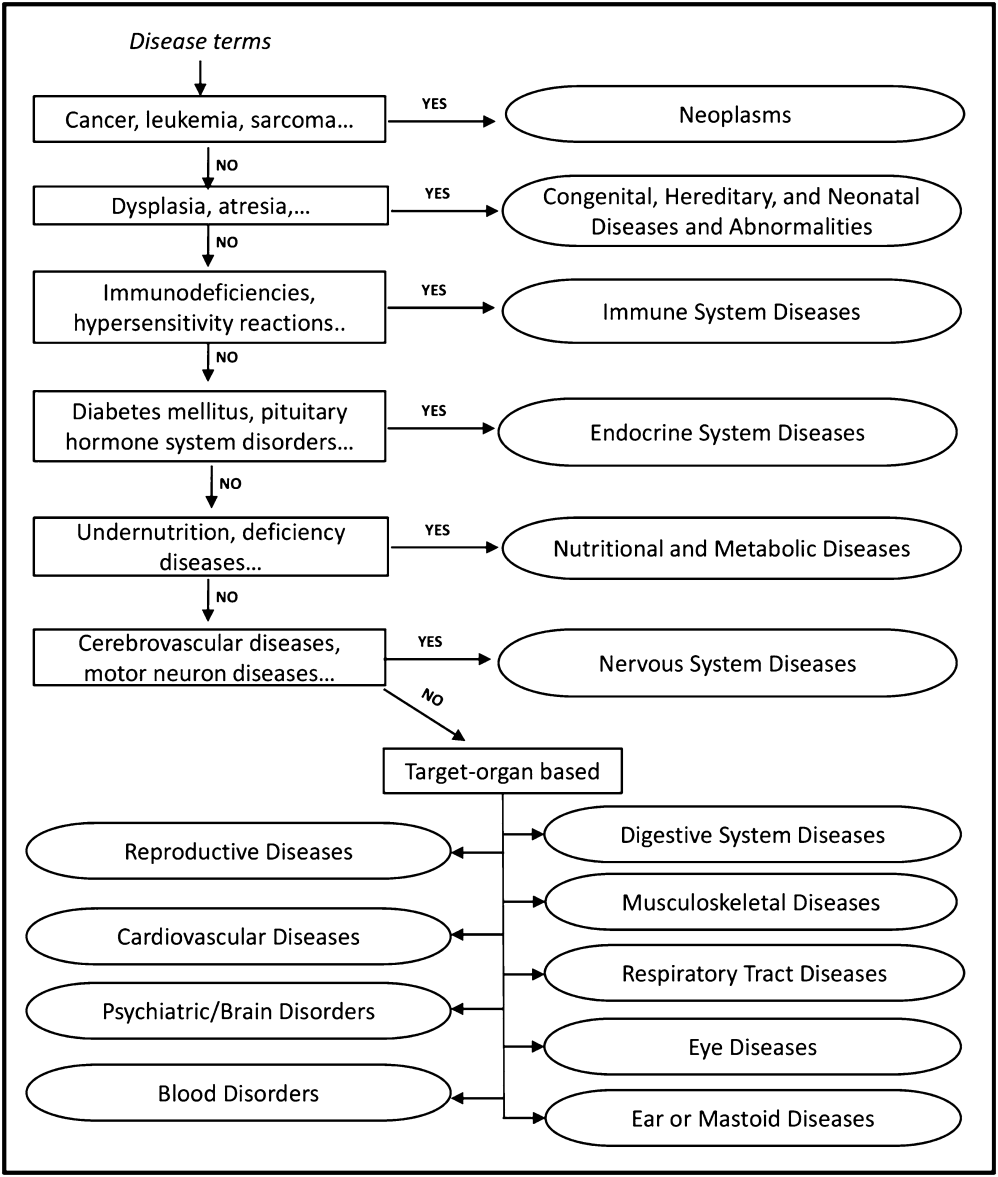
ICD-11 based rules for non-redundant categorization of disease terms. Ovals represent retitled parent disease terms.

Many of the disease terms in DisGeNET^69^ are mapped to multiple MeSH^58^ headings. E.g. ovarian neoplasm is linked to neoplasms and reproductive disorders. An empirical rule-based method based on ICD-11^79^ classification (Fig. 4) was adopted to uniquely categorize the disease terms at the parent level.

For complete documentation of merged terms refer to Supplementary Table S1.

### Tools

#### Comorbidity analysis

For a pair of diseases (${D}_{i}$_,_ ${D}_{j}$), the list of PCOS-associated genes was retrieved from the gene-disease dataset of PCOSKB_R2_ (see “Curation of the gene-disease association dataset” section). Four different algorithms have been used to predict the risk of comorbidity in women with PCOS. The comorbidity scores are illustrated as dynamic heat maps created using AnyChart JS^80^ package.

##### Based on shared genes

This method is based on the principle that disease relationships are dependent on their shared genes^81^. A score to predict the risk of diseases ${D}_{i}$ and ${D}_{j}$ to co-occur is calculated using the below equation

$$Comorbidity_{shared genes} \left( {D_{i} ,D_{j} } \right) = \left[ {\frac{{\left( {G_{{D_{i} }} \cap G_{{D_{j} }} } \right)}}{{min\left( {G_{{D_{i} }} , G_{{D_{j} }} } \right) }} } \right] \times 100$$

where $G_{{D_{i} }}$ and $G_{{D_{j} }}$ are PCOS genes associated with diseases $D_{i}$ and $D_{j}$.

The score is directly proportional to the number of shared genes; hence a higher score indicates a higher risk of comorbidity.

##### Based on the uniqueness of shared genes

This method is based on the observation that diseases, whose genes are not associated with multiple diseases, have a higher comorbidity risk as compared to diseases caused by genes associated with multiple diseases^82^.

The uniqueness of *i*th gene ‘$g_{i}$’ associated with diseases $D_{i}$_,_ $D_{j}$ is calculated as:

$$Uniqueness \left( {g_{i} } \right) = \left[ {1 - \sqrt {\frac{{D_{{g_{i} }} }}{{D^{T} }}} } \right]$$

where $D^{T}$ represents the total number of diseases in the gene-disease dataset and $D_{{g_{i} }}$ is the number of diseases associated with $i{\text{th}}$ gene.

If $n genes \in D_{i} \cap D_{j}$ then, comorbidity of each disease pair is calculated as follows:

$$Comorbidity_{uniqueness} \left( {D_{i} ,D_{j} } \right) = \mathop \sum \limits_{n = 1}^{n} \left[ {Uniqueness\left( {g_{i} } \right)} \right]_{n}$$

The score is directly proportional to the number of uniquely shared genes, hence a higher score indicates a higher risk of comorbidity for the pair of diseases.

##### Based on the biological process and molecular function of associated genes

This algorithm is based on the inference that 95% of disease links can be predicted by the functional overlap of the associated genes^81^. Disease pair comorbidity risk is calculated and scored as per the standard Jaccard index^83^.

$$Comorbidity_{ontology} \left( {D_{i} ,D_{j} } \right) = \left[ {\left| {\frac{{GO_{i} \cap GO_{j} }}{{GO_{i} \cup GO_{j} }}} \right| } \right] \times 100$$

where $GO_{i}$ and $GO_{j}$ are the set of distinct molecular functions and biological processes for genes of diseases *i* and *j* respectively as retrieved from Gene Ontology (GO) database.

The score is directly proportional to the functional overlap of disease-associated genes and therefore higher score indicates a higher risk of comorbidity for the pair of diseases.

##### Based on network separation of disease genes in the human interactome

Diseases whose genes are located closer in the human interactome have a higher probability of co-occurrence as compared to diseases with genes spread apart in the network^84^. Experimentally validated human protein–protein interactions from STRING v11^68^ were used for the algorithm. The comorbidity score is calculated as:

$$Comorbidity_{Shortest\;path} \left( {D_{i} ,D_{j} } \right) = D_{ij} - \frac{{D_{ii } + D_{jj} }}{2}$$

where $D_{ii}$ and $D_{jj}$ is the average of minimum distances of each gene associated with disease *i* and *j* respectively and $D_{ij}$ is the average of minimum distances between genes of diseases *i* and *j*.

Since the score represents the network-based separation of disease-associated genes, a lower score indicates higher risk of comorbidity for the pair of diseases.

#### Network analysis

This tool can be used for visualization of disease networks, identification of enriched pathways, and prioritization of disease genes. Vis.js^85^ visualization library was used for dynamic network creation and visualization. The tool has three modules as described below.

##### Disease-disease network

A dynamic subset of the human disease network^86^ can be created for a selected group of diseases. Diseases are represented as nodes and the size of a node is proportional to the number of genes or miRNAs associated with the disease. Disease nodes are connected by edges based on the number of shared genes or miRNAs between them. Users can select multiple diseases for the identification of enriched pathways in these diseases.

##### Pathway enrichment analysis

The disease-pathway associations are inferred based on mapping disease-associated genes and target genes of associated miRNAs to their pathways^87^. Enriched pathways are identified based on hypergeometric distribution with the threshold *p* value set as 0.05 (gene dataset) and 0.001(miRNA dataset) based on the data size. Users can select pathways and visualize the network. Each pathway is represented as a node and is connected to other pathways in the network based on common genes or miRNAs. The thickness of the edge is proportional to the number of shared genes or miRNAs. If gene dataset is selected then, the enriched pathways can be examined for the identification of critical hub and bottleneck genes through the *Gene network analysis* module.

##### Gene network analysis

Experimentally validated interactions from STRING v11^68^ were used for creating gene interaction networks for enriched pathways. Critical genes in these pathways were identified based on network topological properties such as degree, closeness centrality, and betweenness centrality calculated using graph package in R^88^. The hub and bottleneck genes were defined based on the study of Rakshit et al.^89^.

Hub genes: *Degree* > (*Mean of Degree* + (2* Standard Deviation)) OR *Closeness centrality* > (*Mean of closeness centrality* + (2* *Standard Deviation*)).

Bottleneck genes: *Degree* < (*Mean of Degree*) AND *Betweenness centrality* > (*Mean of Betweenness centrality*).

#### Venn analysis

The common and unique list of genes, pathways, and ontologies can be identified for a selected list of diseases using this tool. jvenn source code ^16^ was used to develop the interactive 6-way Venn diagram.

## Supplementary information

Supplementary Information 1.

Supplementary Information 2.

## Data Availability

The data can be accessed and downloaded from the PCOSKB_R2_ portal (http://www.pcoskb.bicnirrh.res.in/).
