# Supplementary material for: PCOSKBR2: a database of genes, diseases, pathways, and networks associated with polycystic ovary syndrome
Source: Sci Rep. 2020 Sep 7;10:14738. doi: 10.1038/s41598-020-71418-8 (PMC7477240; doi:10.1038/s41598-020-71418-8)
Supplement: Supplementary file 1 — Supplementary Information 1. [file 41598_2020_71418_MOESM1_ESM.docx]

**SUPPLEMENTARY DATA**

**PCOSKB_R2_: a database of genes, diseases, pathways, and networks associated with polycystic ovary syndrome**

Mridula Sharma^1Ɨ^, Ram Shankar Barai^1Ɨ^, Indra Kundu^1Ɨ^, Sameeksha Bhaye^1^, Khushal Pokar^1^, Susan Idicula-Thomas^1^*

^1^ Biomedical Informatics Center, Indian Council of Medical Research-National Institute for Research in Reproductive Health, Mumbai- 400012, India

^Ɨ^ The authors wish it to be known that, in their opinion, the first three authors should be regarded as Joint First Authors.

*****To whom correspondence should be addressed:

Dr. Susan Idicula-Thomas, E-mail: [thomass@nirrh.res.in](mailto:thomass@nirrh.res.in), Tel: 91**-** 2224192107/04

**Supplementary Figure:**

**Fig. S1.** Identification of enriched pathways, hub genes and tissue-specific interactions of hub genes for a) diabetes and hypertension, and b) anxiety and depression.
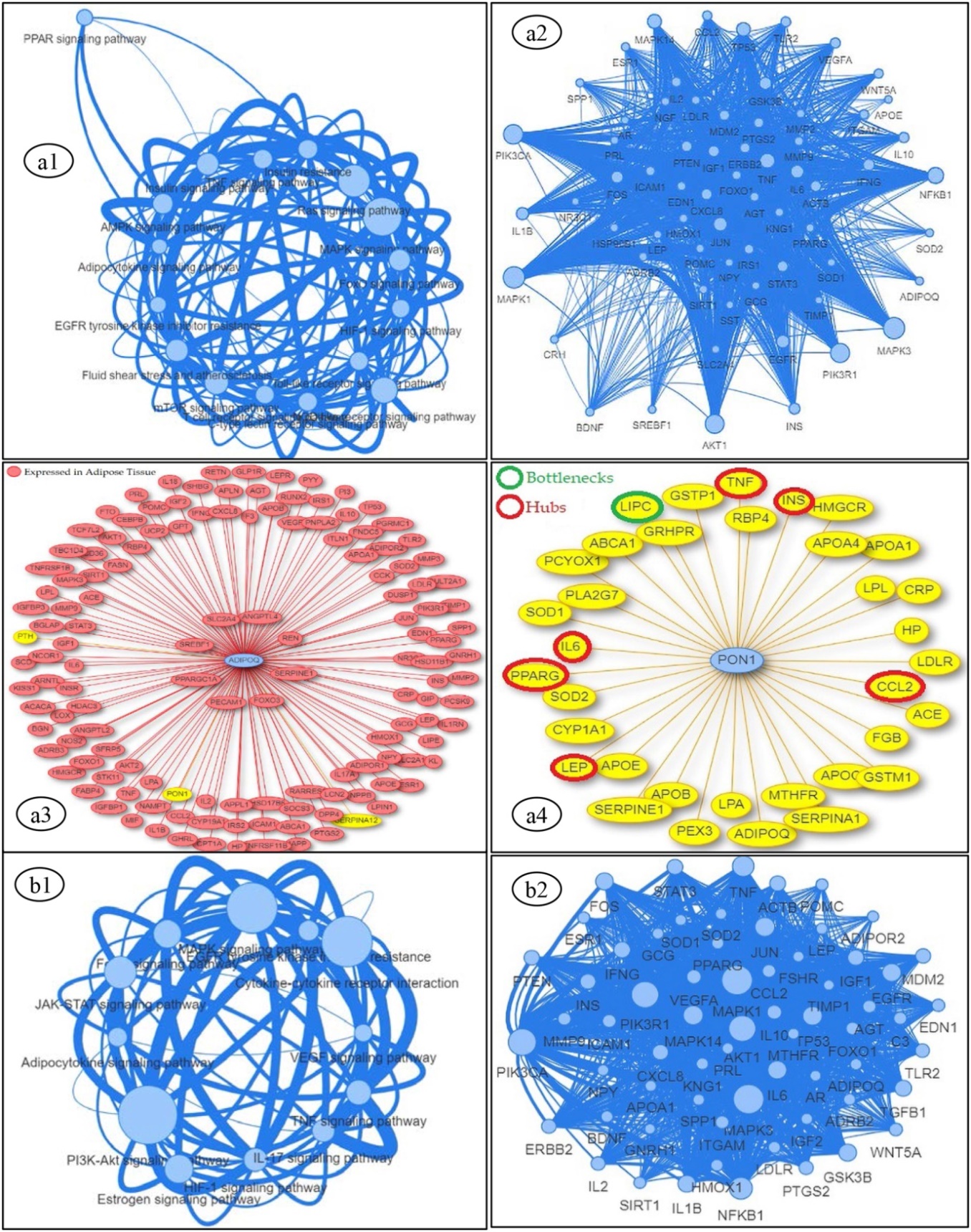


**Supplementary Tables:**

**Supplementary Table S1: Given as an excel file “Supplementary Table S1.xlsx”**

**Supplementary Table S2.** Enriched pathways and associated genes in mental disorders

| Mechanism | Pathway names | *Genes* | p-value |
| --- | --- | --- | --- |
| Insulin resistance | MAPK signaling pathway | *MAPK14, DUSP1, EGFR, AKT1, FOS, IGF1, IL1A, IL1B, INS, ATF4, NGF, MAPK3, BDNF, TGFB1, TNF, TNFRSF1A, VEGFA* | 0.0002 |
|  | FoxO signaling pathway | *MAPK14, EGFR, AKT1, IGF1, IL6, IL10, INS, MAPK3, SOD2, STAT3, TGFB1* | 0.004 |
|  | PI3K-Akt signaling pathway | *EGFR, AKT1, GH1, GSK3B, IGF1, IL2, IL6, INS, ATF4, NGF, MAPK3, PRL, BDNF, BRCA1, VEGFA* | 0.0021 |
| Obesity | Cytokine-cytokine receptor interaction | *GH1, IFNG, IL1A, IL1B, IL2, IL6, CXCL8, IL10, IL18, CXCL10, LEP, LIF, NGF, PRL, CCL2, TGFB1, TNF, TNFRSF1A, TNFRSF1B* | 1.3E-5 |
|  | JAK-STAT signaling pathway | *EGFR, AKT1, GH1, IFNG, IL2, IL6, IL10, LEP, LIF, PRL, STAT3* | 0.0004 |
|  | Adipocytokine signaling pathway | *AKT1, LEP, NPY, POMC, STAT3, TNF, TNFRSF1A, TNFRSF1B* | 0.0287 |
| Altered androgen levels | Estrogen signaling pathway | *EGFR, AKT1, ESR1, ESR2, FOS, ATF4, POMC, MAPK3* | 0.023 |
|  | SUMOylation of intracellular receptors | *ESR1, NR3C1, AR* | 0.049 |
|  | Renin secretion | *ADRB3, ACE, AGT, REN* | 0.0489 |
| HPA axis abnormalities | HIF-1 signaling pathway | *EGFR, AKT1, HIF1A, IFNG, IGF1, IL6, INS, NOS2, SERPINE1, MAPK3, STAT3, VEGFA* | 0.0006 |
|  | Regulation of gene expression by Hypoxia-inducible Factor | *HIF1A, VEGFA* | 0.025 |
| Inflammation | TNF signaling pathway | *MAPK14, AKT1, FOS, IL1B, IL6, CXCL10, LIF, ATF4, MAPK3, PTGS2, CCL2, TNF, TNFRSF1A, TNFRSF1B* | 4.3E-5 |
|  | IL-17 signaling pathway | *MAPK14, FOS, GSK3B, IFNG, IL1B, IL6, CXCL8, CXCL10, MAPK3, PTGS2, CCL2, TNF* | 0.00018 |

**Supplementary Table S3:** Statistics summary for the manually curated dataset in PCOSKB_R2_

| **Content** | | **Genes** | **miRNAs** |
| --- | --- | --- | --- |
| Manually curated | | 533 | 29 |
| Genes | *Summary* | 497 | 29 |
|  | *RefSeq mRNA* | 367 | - |
|  | *Ensemble gene* | 522 | - |
| Proteins | *Protein* | 517 | - |
|  | *Function* | 442 | - |
|  | *Structure* | 359 | - |
| Ontologies | *Biological process* | 203 | - |
|  | *Cellular component* | 153 | - |
|  | *Molecular function* | 188 | - |
| SNPs | *SNP* | 64 | 2 |
|  | *Sequence* | 45 | - |
|  | *Function* | 47 | - |
| Interactions | *STRING* | 488 | 25 |
|  | *MINT* | 312 | 15 |
|  | *IntAct* | 474 | 25 |
| Pathways | *KEGG* | 407 | 17 |
|  | *Reactome* | 457 | - |
| Diseases | *PubMed* | 55 | 2 |
|  | *DisGeNET* | 474 | 24 |
